# Psychological Factors Are Associated With Lower Limb Kinematics During Uphill Walking and Running in Women With Patellofemoral Pain

**DOI:** 10.1002/pri.70208

**Published:** 2026-04-07

**Authors:** Isabela Regina dos Reis, Ana Caroline Brites de Magistris Martins, Beatriz Paiva Soares, Jamilly Radiuc Cipolli, Deborah Hebling Spinoso

**Affiliations:** ^1^ Laboratory of Biomechanics. Faculty of Philosophy and Sciences São Paulo State University (UNESP) Marília São Paulo Brazil; ^2^ Physical Therapy and Occupational Therapy Department Faculty of Philosophy and Sciences São Paulo State University (UNESP) Marília São Paulo Brazil

**Keywords:** fear of movement, functionality, knee pain

## Abstract

**Background and Purpose:**

Patellofemoral pain (PFP) is a common condition in women and is associated with high recurrence rates. Kinematic alterations are among the factors that may persist even after muscle strengthening programs. Given the multifactorial nature of PFP, non‐mechanical factors, such as kinesiophobia, may be related to the movement patterns adopted by this population. This study aimed to investigate the associations between kinesiophobia, pain‐related factors, and lower limb kinematics during uphill walking and treadmill running in women with PFP.

**Methods:**

This was a cross‐sectional study. Data were collected from 22 young women (24.0 ± 5.87 years) with PFP. Anthropometric data were collected, and participants completed the Tampa Scale for Kinesiophobia, the Pain Catastrophizing Scale, and the Numerical Rating Scale for pain. Subsequently, kinematics of the affected lower limb was analyzed during uphill walking and treadmill running. The angles of knee flexion, hip flexion, ankle dorsiflexion, knee valgus, and pelvic tilt were calculated. Pearson's correlation coefficient was used for statistical analysis, with the significance level set at *p* < 0.05.

**Results:**

Significant negative correlations were observed between knee flexion angle and kinesiophobia (*r* = −0.381, *p* = 0.040) as well as pain catastrophizing (*r* = −0.482, *p* = 0.023) during uphill walking. During treadmill running, stronger negative correlations were found between knee flexion angle and both kinesiophobia (*r* = −0.694, *p* = 0.008) and pain catastrophizing (*r* = −0.972, *p* = 0.008). No significant correlations were observed for the other joint angles analyzed.

**Discussion:**

The findings of this study indicate that higher levels of kinesiophobia and pain catastrophizing are associated with reduced knee flexion during uphill walking and treadmill running in women with PFP. Rather than suggesting a causal effect, these results highlight a meaningful relationship between psychological factors and movement patterns during functional activities. Reduced knee flexion may reflect a protective or avoidance strategy commonly observed in individuals with elevated fear of movement or maladaptive pain‐related beliefs. Clinically, these associations underscore the importance of considering psychological factors during assessment and rehabilitation, as they may be related to altered movement strategies and functional performance in individuals with knee pain.

## 1 Introduction

Patellofemoral pain (PFP) is characterized by pain in the anterior region or around the knee, which can be aggravated by activities that involve overloading or compressing the patellofemoral joint, including squatting, jumping, running, and going up and down the steps (Gaitonde et al. 2019). The prevalence of PFP is higher in women, who are 2.23 times more likely to develop PFP, than men (Boling et al. 2010).

Patellofemoral pain (PFP) is recognized as a multifactorial and complex condition that cannot be fully explained by biomechanical alterations alone (Bazett‐Jones et al. 2023; Gilgallon et al. 2024; Fernades et al. 2025; Gragnani et al. 2025). Recent literature emphasizes that both physical and non‐physical factors — including biomechanical, psychosocial, and lifestyle components — contribute to the presentation and persistence of PFP symptoms. For instance, Gragnani et al. (2025) argue that PFP involves a complex interplay of influences and advocate for a multidisciplinary approach to its assessment and management, highlighting the limitations of exclusively biomechanical explanations. In parallel, research has demonstrated relationships between psychological factors and physical activity behaviors, indicating that psychological constructs such as fear of movement and pain‐related cognitions may be interrelated with how individuals engage in physical tasks. Together, this body of evidence supports the notion that psychological factors warrant consideration alongside mechanical variables when investigating movement patterns in women with PFP. Current scientific guidelines support that exercise‐based treatment is effective for short‐term symptom improvement; however, unfavorable outcomes after treatment have been reported in more than 50% of PFPD patients (Lankhorst et al. 2016; Willy et al. 2019).

The role of biomechanics has been proposed as one of the factors contributing to the persistence of symptoms in people with PFP, given that kinematic changes persist after treatment (Bazett‐Jones et al. 2023; Martins et al. 2022). Studies have shown that women with PFP walk with a lower range of dorsiflexion and hip and knee flexion when compared to controls (Martins et al. 2022). Xie et al. (2023) reported higher hip adduction during running and unipodal squatting in women with PFP compared to asymptomatic controls. Greater pelvic drop was observed during step descent and unipodal squatting (Nakagawa et al. 2012). These kinematic changes have been identified as a protective mechanism to avoid excessive stress on the patellofemoral joint in an attempt to reduce pain (Brechter and Powers 2002; Powers et al. 2017). A study by Rabelo and Lucarelli (2018) showed that performing resistance exercises during rehabilitation to improve the muscle strength of the hip and knee extensors and hip abductors did not modify the kinematic movement pattern observed in women with PFP.

In this context, a better understanding of the factors related to kinematic changes in movement in people with PFP has been sought. Silva et al. (2019) reported that kinesiophobia has a bigger influence on kinematic changes than lower limb muscle strength. Kinesiophobia is understood as the fear associated with movement, due to the belief that movement can increase pain (activity avoidance focus) and that pain is a sign of bodily damage (somatic focus) (Pazzinatto et al. 2020). Kinesiophobia has recently been associated with pain intensity and functional disability in people with PFP (Doménech et al. 2014; Maclachlan et al. 2018; Priore et al. 2019). The relationship between kinesiophobia and movement kinematics in people with PFP is still unclear. So far, only two studies have investigated this relationship. Silva et al. (2019) reported that women with PFP with kinesiophobia had less knee flexion during stair descent. Vasconcelos et al. (2023) showed that kinesiophobia is related to hip internal rotation and knee flexion during unipodal jumping.

Understanding how fear of movement can contribute to persistent kinematic changes in women with PFP can help guide treatment strategies for people with PFP, in order to obtain more favorable results in the medium and the long term. In this regard, the aim of this study was to investigate the relationship between kinesiophobia and lower limb kinematics during uphill walking and running in women with PFD. The hypothesis of this study is that women with higher kinesiophobia will have lower knee flexion, ankle dorsiflexion, hip flexion, higher pelvic drop and higher knee valgus during uphill walking and running.

## 2 Methods

### 2.1 Participants

This is a cross‐sectional study involving 22 volunteers with PFP, aged 24.00 ± 5.87 years. The eligibility criteria for inclusion of individuals with PFP were women aged 18‐–45 years; anterior knee pain in at least two of the following activities—going up/down stairs, squatting, running, or jumping; insidious onset of pain for at least four months; and having pain listed out in at least 3 on the Numerical Rating Scale (NRS). The exclusion criteria were as follows: having had knee joint surgery, meniscal and/or ligament injuries, patellar instability, and cardiovascular, congenital, or musculoskeletal disorders that could affect the evaluations (Silva et al. 2019). The project was approved by Ethics Committee (5.690.291), and all the participants were properly informed of the objectives of the study, and they signed the informed consent form.

### 2.2 Assessment Procedures

The assessment procedures were conducted on a single day at the Biomechanics Laboratory. Initially, anthropometric data were collected and the Tampa Scale for Kinesiophobia, Pain Catastrophizing Scale, and Numerical Rating Scale were used. Subsequently, a kinematic assessment of the lower limb was carried out during uphill walking and running.

#### 2.2.1 Tampa Scale for Kinesiophobia

The Tampa Scale for Kinesiophobia consists of a 17‐statement questionnaire designed to measure the level of fear associated with movement and the possibility of re‐injury as a result of physical activity. The participants must indicate their degree of agreement or disagreement with each sentence by selecting one among the points on a scale of one to four. The total score can range from 17 to 68, with a higher value (68) indicating a greater fear of injury recurrence and kinesiophobia (Priore et al. 2020).

#### 2.2.2 Pain Catastrophizing Scale

The Pain Catastrophizing Scale is a 13‐item questionnaire that describes the thoughts and feelings that individuals experience when they are in pain. Participants were asked to reflect on their past experiences related to pain and to select one of the 13 thoughts or feelings that best represented their current perception of pain. The scale score ranges from 0 to 52, with 52 indicating an intense tendency to catastrophize pain, that is, an overly negative and pessimistic perception of the painful experience (Priore et al. 2020).

#### 2.2.3 Numerical Rating Scale

The Numerical Rating Scale (NRS) is a visual representation in the form of a straight line, containing numbers from 0 to 10, where each number corresponds to a specific level of pain, ranging from no pain (0) to extreme pain (10). The patient is asked to select the number that most adequately describes their current pain.

#### 2.2.4 Kinematic Analysis

**FIGURE 1:**
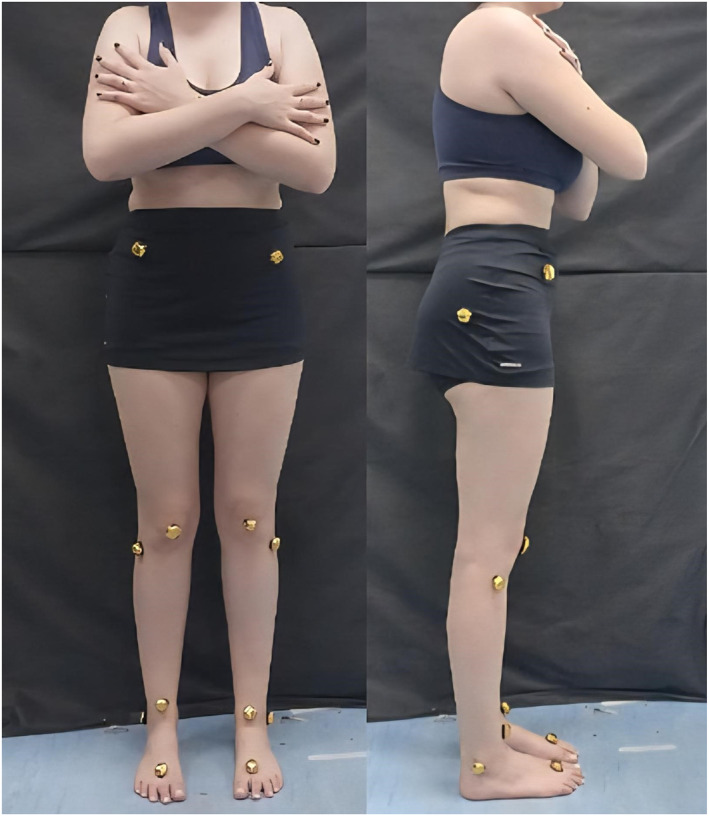
Placement of photoreflective markers on anatomical landmarks.

Two cameras (model DCR‐SR68, Sony Corporation, CHN) with a sampling frequency of 60 Hz were used to capture movement in the frontal and sagittal planes. The cameras were positioned 77 cm above the ground using tripods (Kingston et al. 2020). Photoreflective markers were fixed to the lower limb affected by PFP, and in the case of bilateral involvement, the limb with the most symptoms was included. The anatomical points used were anterosuperior iliac spine bilaterally, greater trochanter of the femur, lateral condyle of the femur, midpoint of the patella, lateral malleolus, midpoint between the malleoli, and dorsal region between the first and second metatarsals. Figure 1 illustrates the positioning of the photoreflective markers.

Initially, the volunteers walked on the treadmill (Evolution Fitness) at their chosen speed for 5 min to warm up. The examiner then increased the incline of the treadmill to 10% and the volunteers walked for 3 min. For the running task, the volunteers walked on the treadmill at their chosen speed, while the examiner gradually increased the treadmill speed to 9.3 Km/h, where the volunteers were instructed to run for 3 min. The running speed was parameterized to allow comparison of the findings with other studies in the literature (Noehren, Pohl, et al. 2012; Noehren, Sanchez, et al. 2012). All participants remained barefoot during data collection. The order of the tasks was randomized and there was a two‐minute rest between them.

#### 2.2.5 Data Analysis

**FIGURE 2:**
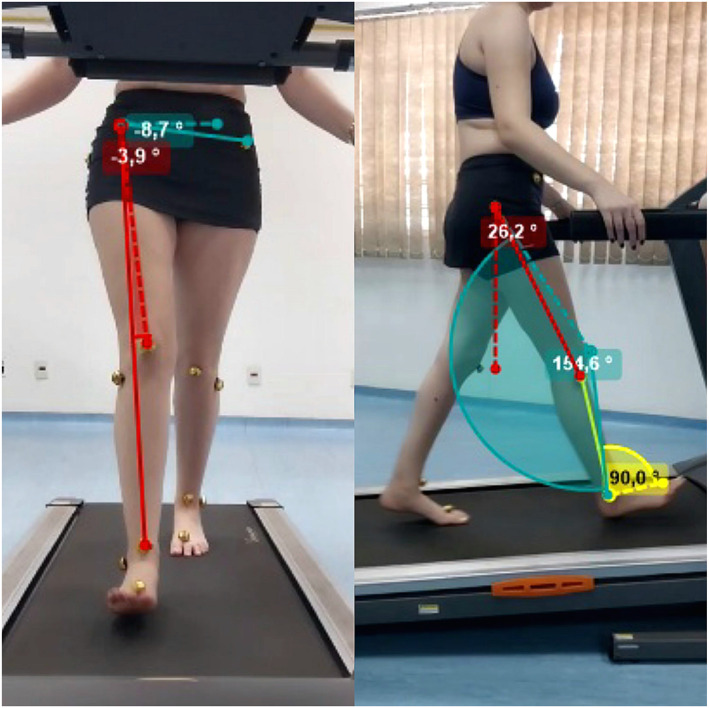
Kinematic analysis of joint angles during the initial stance phase of uphill walking.

The data were processed using Kinovea software (Morita and Navega 2023). The angles of hip flexion, knee flexion, ankle dorsiflexion, hip internal rotation and pelvic tilt were calculated, as shown in Figure 2. The average of six steps was calculated for data analysis.

### 2.3 Statistical Analysis

Statistical analysis was carried out using PASW Statistics 18.0 (SPSS) software. Descriptive analysis was carried out for the variables analyzed. Pearson's correlation test was applied to verify the association between the variables. The correlation classification was defined as *r* = 0.8–1 very high; 0.6–0.79 high; 0.40–0.59 moderate; 0.20–0.39 low; < 0.20 very low. The significance level adopted was *p* < 0.05.

## 3 Results

The anthropometric characteristics of the participants are described in Table 1. The descriptive characteristics of the lower limb kinematics during uphill walking and treadmill running are shown in Table 2.

**TABLE 1:** Description of the study participants.

	Average ± standard deviation
Age (years)	24.00 ± 5.87
Body Mass (kg)	63.8 ± 12.62
Height (m)	1.64 ± 0.06
BMI (kg/m^2^)	23.65 ± 4.21
Current pain level (NRS)	4.00 ± 1.27

Abbreviation: NRS, Numerical Rating Scale.

**TABLE 2:** Kinematic variables during uphill walking and running and with kinesiophobia scores.

	Average ± standard deviation
Kinematics walk uphill (°)	
Hip flexion	28.03 ± 4.99
Pelvic tilt	3.31 ± 2.24
Knee flexion	30.45 ± 7.65
Knee valgus	3.5 ± 2.42
Ankle dorsiflexion	7.76 ± 6.01
Running kinematics (°)	
Hip flexion	21.54 ± 3.25
Pelvic tilt	2.7 ± 1.46
Knee flexion	34.03 ± 5.3
Knee valgus	2.91 ± 1.86
Ankle dorsiflexion	6.95 ± 5.53
Kinesiophobia	
Numerical rating scale	4.00 ± 1.27
Tampa scale	37.77 ± 6.84
Pain catastrophizing	20.09 ± 9.64

The correlation coefficients between the kinematic variables and the kinesiophobia scales are shown in Table 3. There was a weak and moderate significant negative correlation between the knee flexion angle during uphill walking and the score on the kinesiophobia scale (*r* = −0.381 *p* = 0.040) and the pain catastrophizing scale (*r* = −0.482 *p* = 0.023), respectively. During the treadmill running activity, there was a significant high and very high negative correlation between the knee flexion angle and the score on the kinesiophobia (*r* = −0.694 *p* = 0.008) and pain catastrophizing (*r* = −0.972 *p* = 0.008) scales, respectively. There was no correlation for the other joint angles assessed.

**TABLE 3:** Correlation coefficient between kinesiophobia, pain catastrophizing and kinematics of uphill walking and running.

	Tampa scale for kinesiophobia	Catastrophizing pain scale
Walking uphill		
Hip flexion	−0.191 *p* = 0.394	−0.249 *p* = 0.290
Pelvic tilt	0.191 *p* = 0.369	0.333 *p* = 0.130
Knee flexion	**−0.381 *p* = 0.040**	**−0.482 *p* = 0.023**
Knee valgus	0.293 *p* = 0.396	0.052 *p* = 0.828
Ankle dorsiflexion	−0.073 *p* = 0.746	−0.243 *p* = 0.302
Running		
Hip flexion	−0.193 *p* = 0.414	−0.323 *p* = 0.143
Pelvic tilt	0.030 *p* = 0.895	0.154 *p* = 0.493
Knee flexion	**−0.694 *p* = 0.008**	**−0.972 *p* = 0.008**
Knee valgus	0.244 *p* = 0.299	0.003 *p* = 0.889
Ankle dorsiflexion	−0.037 *p* = 0.878	−0.058 *p* = 0.796

## 4 Discussion

The present findings demonstrate that higher levels of kinesiophobia and pain catastrophizing are associated with reduced peak knee flexion during uphill walking and treadmill running in women with patellofemoral pain. In contrast, no significant associations were observed for the other joint angles analyzed. These results reinforce the relevance of psychological factors in understanding movement behavior during functional tasks in this population.

Kinesiophobia has been associated with worse pain and functional disability and may be related to task‐dependent changes in movement patterns (Silva et al. 2019). In the present study, both activities were associated with kinesiophobia; however, stronger correlations were observed during treadmill running compared with uphill walking. Running imposes higher joint loads, which may increase the relevance of fear‐related movement adaptations. These findings suggest that the mechanical demands of the task may modulate the strength of the association between psychological factors and biomechanical behavior.

The primary kinematic characteristic associated with psychological factors in this study was reduced knee flexion. This finding is consistent with previous studies reporting associations between higher levels of kinesiophobia and lower peak knee flexion during functional tasks such as stair descent and unipodal jumping (Silva et al. 2019; Vasconcelos et al. 2023). Reduced knee flexion may reflect a protective or avoidance strategy aimed at limiting patellofemoral joint loading and pain exacerbation. As patellofemoral joint reaction forces increase with greater knee flexion angles, this strategy may be adopted to reduce joint contact pressure and discomfort (Hart et al. 2022). However, reducing knee flexion during load‐bearing tasks may compromise shock absorption and increase ground reaction forces acting on the knee, potentially contributing to unfavorable mechanical consequences over time (Silva et al. 2015).

No significant associations were observed between kinesiophobia and hip, ankle, or pelvic kinematics in the present study. This may be related to the specific motor demands of uphill walking and treadmill running, which may not elicit the same compensatory strategies observed in more challenging tasks such as single‐leg squatting, jumping, or stair descent. Previous studies have reported greater pelvic drop, hip adduction, ankle dorsiflexion, and hip flexion in women with PFP during more demanding tasks (Nakagawa et al. 2012; Xie et al. 2023), whereas others have found no significant differences in trunk and pelvis kinematics during walking and running (Haghighat et al. 2021; Noehren, Pohl, et al. 2012; Noehren, Sanchez, et al. 2012), supporting the task‐dependent nature of kinematic alterations.

Although most studies examining psychological factors and movement patterns in patellofemoral pain are cross‐sectional, an important prospective study demonstrated that physical function and fear of movement were not associated with the risk of developing patellofemoral pain in young women. Fear of movement may therefore develop as a consequence of persistent patellofemoral pain rather than representing a risk factor or a defining characteristic in women with a shorter symptom duration (Pazzinatto et al. 2023). A review by Kim et al. (2023) demonstrated that changes in hip and knee strength are not associated with improved clinical outcomes following rehabilitation in individuals with patellofemoral pain. This finding suggests that although muscular strength is an important factor for functionality, the cause of pain is not purely biomechanical. Therefore, it is necessary to consider other factors in the management of individuals with patellofemoral pain, such as pain perception and psychological aspects. A more comprehensive and multifactorial approach may therefore be essential to optimize rehabilitation outcomes in this population.

Rethman et al.’s meta‐analysis found that higher levels of kinesiophobia are moderately associated with poorer self‐reported function and weakly associated with pain across PFP cohorts, reinforcing the clinical relevance of psychological factors in symptoms and functional limitations (Rethman et al. 2023). Furthermore, Gilgallon et al. (2024) observed that pain self‐efficacy is moderately associated with physical activity levels among individuals with PFP, highlighting that psychological constructs can relate meaningfully to broader activity behaviors beyond joint kinematics. The study by Priore et al. (2020) demonstrated that the use of a knee brace is effective in reducing kinesiophobia at both 2 and 6 weeks in individuals with patellofemoral pain. The incorporation of strategies aimed at reducing kinesiophobia may enhance the effectiveness of interventions for individuals with patellofemoral pain, potentially leading to greater treatment adherence, reduced pain, and improved functional outcomes.

Although causal relationships cannot be established from the present findings, the observed associations are consistent with this body of evidence and support the inclusion of psychological factors in explanatory and biopsychosocial models of patellofemoral pain. This challenges the traditional biomechanical paradigm that prioritizes strengthening as the primary intervention strategy. Moreover, kinematic alterations have been shown to persist even after rehabilitation and may contribute to recurrent pain episodes (Martins et al. 2022). Together, these findings suggest that non‐mechanical factors, such as kinesiophobia and maladaptive pain‐related beliefs, may play an important role in movement behavior and symptom persistence.

In this context, interventions targeting psychological and behavioral aspects of patellofemoral pain may be important adjuncts to exercise‐based rehabilitation. Overall, the present findings support a biopsychosocial approach to patellofemoral pain, in which psychological factors are considered alongside biomechanical and clinical variables to optimize assessment and rehabilitation strategies.

This study has some limitations. It has a cross‐sectional design, and therefore, it is not possible to establish a causal relationship between the variables. In addition, there was an age group limitation; it did not include adolescents and older women, and therefore, the results could not be extrapolated to other populations.

In summary, kinesiophobia is associated with lower knee flexion joint angulation during uphill walking and treadmill running when it comes to PFP in young women. It is recommended that strategies to reduce kinesiophobia be included in the rehabilitation of women with PFP.

### 4.1 Implications of Physiotherapy Practice

The present findings highlight the clinical relevance of psychosocial factors, particularly kinesiophobia and pain catastrophizing, in influencing lower limb biomechanics during functional and more demanding locomotor tasks. The significant negative correlations observed between the knee flexion angle and both kinesiophobia and pain catastrophizing scores suggest that individuals with higher levels of fear of movement and catastrophic thinking adopt a more protective movement strategy, characterized by reduced knee flexion, especially during treadmill running. From a physiotherapy perspective, these results reinforce the importance of incorporating psychosocial assessment into routine clinical evaluation, particularly in populations with musculoskeletal conditions. The stronger correlations observed during running compared to uphill walking indicate that psychologically driven movement adaptations may become more pronounced as task demand increases. Therefore, functional tasks with higher biomechanical and neuromuscular demands may serve as sensitive indicators of fear‐related movement alterations. Intervention strategies should not focus exclusively on physical impairments but also address maladaptive beliefs and fear‐avoidance behaviors. Cognitive‐behavioral approaches, pain neuroscience education, and graded exposure to movement may be essential components of rehabilitation programs aimed at restoring normal knee kinematics and optimizing functional performance. Additionally, clinicians should be aware that reduced knee flexion during dynamic tasks may increase joint loading and alter shock absorption mechanisms, potentially contributing to symptom persistence or progression of joint dysfunction. Early identification and management of kinesiophobia and pain catastrophizing may therefore play a critical role in preventing long‐term biomechanical maladaptations. Overall, these findings support a biopsychosocial model of care, emphasizing the integration of psychological and biomechanical factors in physiotherapy assessment and intervention planning.

## Author Contributions

**Isabela Regina dos Reis:** conceptualization, data curation, investigation, writing – original draft. **Ana Caroline Brites de Magistris Martins:** data curation, formal analysis and methodology. **Beatriz Paiva Soares:** data curation, formal analysis, writing – review and editing. **Jamilly Radiuc Cipolli:** data curation, formal analysis, methodology, writing – review and editing. **Deborah Hebling Spinoso:** conceptualization, data curation, funding acquisition, project administration, writing – original draft. All authors provided final approval of the version to be submitted.

## Funding

The authors have nothing to report.

## Ethics Statement

The research project was approved by the local Ethics Committee of the Sao Paulo State University (Opinion: 15.690.291).

## Consent

Written consent was obtained from all participants prior to data collection.

## Conflicts of Interest

The authors declare no conflicts of interest.

## Permission to Reproduce Material From Other Sources

The authors have nothing to report.

## Data Availability

The data that support the findings of this study are available from the corresponding author upon reasonable request.
